# Warning about conservation status of forest ecosystems in tropical Andes: National assessment based on IUCN criteria

**DOI:** 10.1371/journal.pone.0237877

**Published:** 2020-08-25

**Authors:** Jin Kyoung Noh, Cristian Echeverria, Janina Kleemann, Hongmi Koo, Christine Fürst, Pablo Cuenca

**Affiliations:** 1 Laboratorio de Cambio Global, Universidad Regional Amazónica Ikiam, Tena, Ecuador; 2 Department Sustainable Landscape Development, Institute for Geosciences and Geography, Martin-Luther-University Halle-Wittenberg Halle, Halle, Germany; 3 Laboratorio de Ecología de Paisaje, Facultad de Ciencias Forestales, Universidad de Concepción, Concepción, Chile; 4 Grupo de Investigación Ecosistemas Tropicales y Cambio Global, Universidad Regional Amazónica Ikiam, Tena, Ecuador; Imperial College London, UNITED KINGDOM

## Abstract

World ecosystems are suffering from anthropogenic and natural pressure. The IUCN (International Union for Conservation of Nature) has developed analogous criteria for the Red List of Threatened Species in order to perform similar risk assessments on ecosystems, creating the Red List of Ecosystems (RLE) methodology. One of the most significant challenges for the construction of these lists is gathering the available information to apply the criteria. By applying IUCN RLE criteria B (the extent of restricted geographic distribution of an ecosystem), we analyzed the threat level of 64 forest ecosystems of the Ecuadorian mainland. According to the results, limited distribution is the key risk to threatened ecosystems, which are associated with anthropogenic pressures. Our study showed that 22% of forest ecosystems are classified as threatened. This evaluation of the forest ecosystem status at a national level could lead to public awareness towards ecosystem conservation and provide reasonable strategies to managers.

## Introduction

Habitat fragmentation is one of the main threats to biodiversity on local, regional and global levels [1]. There is a clear need to manage fragmented ecosystems in order to maintain and conserve the diversity of species as well as ecosystem services [2, 3]. Previously, the majority of efforts to conserve biodiversity have been focused on species, communities or their habitats, but recently, there has been an increasing awareness of the importance of considering larger scales, such as entire ecosystems and landscapes, with the aim of benefiting both biodiversity and human well-being [4–6]. Likewise, the recent tendency in conservation planning is focused on ecosystem-level assessments, which ensures not only the protection of a sufficient portion of all ecosystems within a country but also the persistence of lower-level biodiversity, for example, genetic diversity [7–9].

Despite systematic methods for assessing the threat of extinction of individual species have notably advanced in recent years, there are few widely accepted scientific frameworks for tracking the status of Earth’s ecosystem and identifying those with a high probability of loss or degradation [9–12]. Recognizing this gap, ecosystem-level extinction risk assessments began to be developed and implemented comparable to global standards from the World Conservation Congress in 2008. The IUCN Red List of Ecosystems (RLE) is a newly developed system for assessing the risk of ecosystem collapse, which is designed to evaluate four symptoms of ecosystem degradation: declining distribution, restricted distribution, degradation of abiotic environment and altered biotic processes [13].

An ecosystem is considered under collapse “when it is virtually certain that its defining biotic or abiotic features are lost, and the characteristic native biota is no longer sustained” [14]. A key task is to identify the transition between states either as part of natural variability within an ecosystem type or as a process of collapse and replacement by a different or novel ecosystem type [14]. As land use change is identified as the major driver for biodiversity changes in terrestrial ecosystems [15], this human process potentially contributes to ecosystem collapse. The loss of plant cover has been considered one of the main triggers of degradation, since the structure of the ecosystem is directly involved [16]. For example, Tozer, Leishman [17] used a state-and-transition framework to identify both the processes that drive transitions between different states of a woodland ecosystem and the states that represent ecosystem collapse.

The IUCN provides an effective assessment protocol for establishing a systematic RLE for the world [18–20]. There are five criteria in the risk assessment protocol [21]: criteria A refers to the declining distribution of the ecosystem over a certain period of time (50 years in the past, 50 years in the future, 50 years in any range and historical loss); criteria B refers to ecosystems with a limited geographic distribution; criteria C refers to the degradation of the ecosystem´s abiotic or environmental components over a certain period of time (same as criteria A); criteria D refers to the disruption of biotic processes or interactions fundamental to the ecosystem in a certain period of time (same as criteria A); and criteria E refers to a quantitative analysis that estimates the likelihood of an ecosystem’s collapse. Among these five IUCN RLE criteria, criteria B must compile all the evidence required by the sub-criteria to estimate the extent of occurrence (EOO) and area of occurrence (AOO). Spatially explicit threats (e.g., forest fire, extreme weather events, forest fragmentation, land conversion, and invasion) are considered threats to ecosystem distribution or ecological process decline. In terrestrial ecosystems, literature reviews reveal that temporal trends in the distribution of land use have been proposed and applied as a threat for assessing the status of some types of ecosystems [22, 23]. For example, Rodríguez, Nassar [24] used land cover loss and rate of changes in land cover across multiple spatial scales for an ecosystem risk assessment. On the other hand, because threats may be assessed in at least three dimensions: immediacy, scope, and severity, forest loss also represents the composition, structure and function of the current forest ecosystems. In addition, the combined negative effects and interactions between different drivers of ecosystem collapse must be tested for future conservation action [19].

The tropical Andes range is classified as a center of biodiversity and endemism in the world [25]. The specific studies of ecosystem threats and risk assessments carried out in the tropical Andes were initiated in the late 1980s [26–29]. These studies suggested that the two main threats to ecosystems in the tropical Andes are human land use and forest fragmentation. Despite the ecological importance, the highest deforestation rate has been related to human activities (e.g., logging, agriculture, and grazing) during the last 30 years in this region [30–33]. Recent studies are increasingly worried by the negative effects of forest fragmentation on biodiversity in the tropical Andes [33, 34]. Notwithstanding the growing literature reporting forest decline and land use change driving ecosystem collapse, few studies have assessed conservation status at the ecosystem-level based on the IUCN criteria [35].

Ecuador is home to high-biodiversity terrestrial ecosystems that exhibit very high levels of endemism in the tropics [36]. The tropical Andes of Ecuador is characterized by landscapes with peculiar climatic and topographic conditions where human settlements both affect and depend on natural forest ecosystems [37]. During the last few decades, Ecuador’s native forests have been destroyed, fragmented and associated with anthropogenic disturbances, such as agriculture, logging and grazing [32, 33]. Despite an ongoing trend of forest change (loss and fragmentation), this area still contains a high diversity of forest ecosystems [38].

Against this background, we assessed the conservation status of 64 forest ecosystems of the Ecuadorian mainland. Our analyses provide the first evidence of potential risk of a collapse of forest ecosystems in Ecuador. Considering that many forest ecosystems of the present study are unique, their loss poses significant impacts for biodiversity conservation on a global level. From the conservation point of view, urgent and effective conservation actions may allow the recovery of threatened forest ecosystems located in this biodiversity hotspot.

## Materials and methods

**Fig 1:**
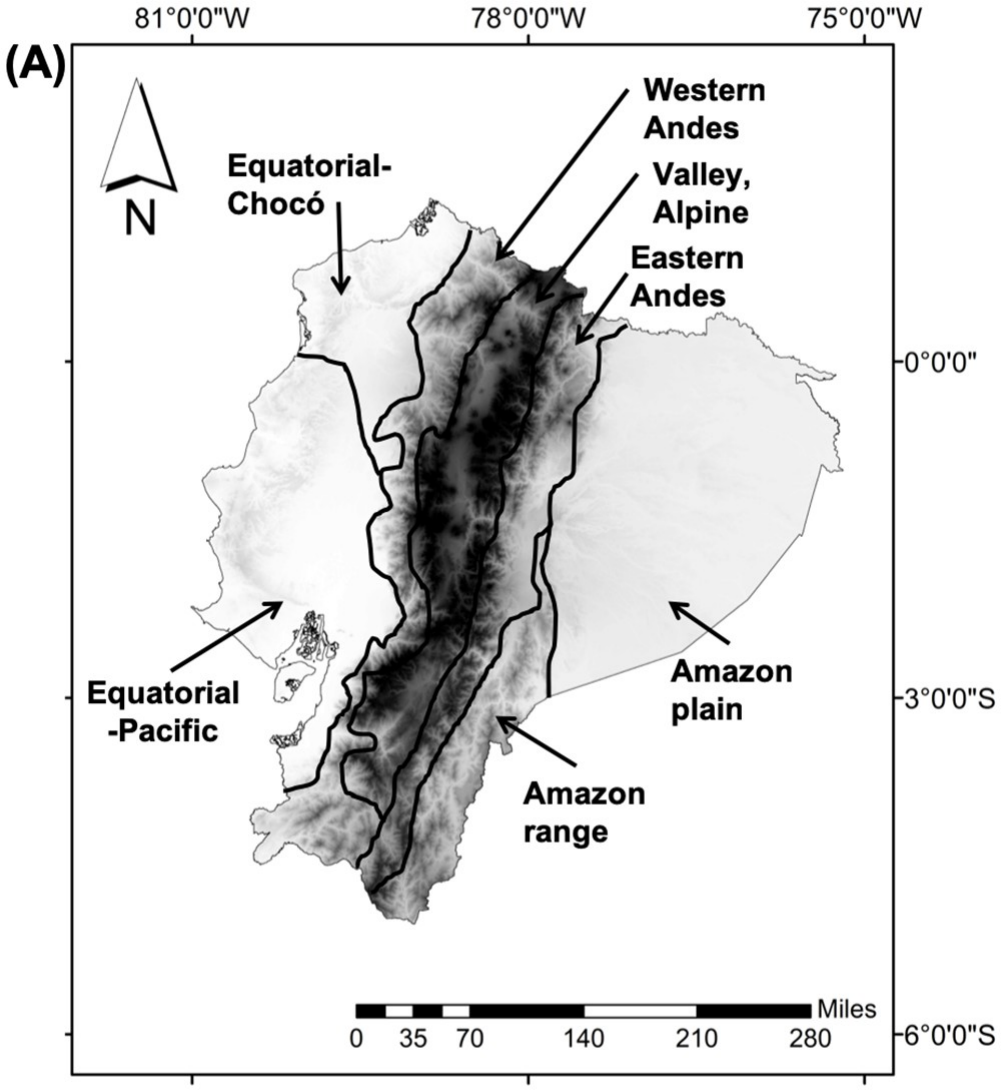
Ecoregion map of Ecuador continent. Elevation detail is shown.

The study was carried out in 64 forest ecosystems equivalent to 54% of national territory (≈ 135,936 km^2^) of the whole Ecuadorian mainland with elevation ranging between 0 and 6000 m a.s.l. (Fig 1). Ecuador is located in a transition zone of two biodiversity hotspots: 1) Choco/Darien western Ecuador and 2) tropical Andes [25]. Likewise, the Ecuadorian Amazon is known as one of the most diverse places on Earth, including a high number of threatened species and regional endemics [39, 40]. Therefore, the study area should obtain conservation priority.

**Fig 2:**
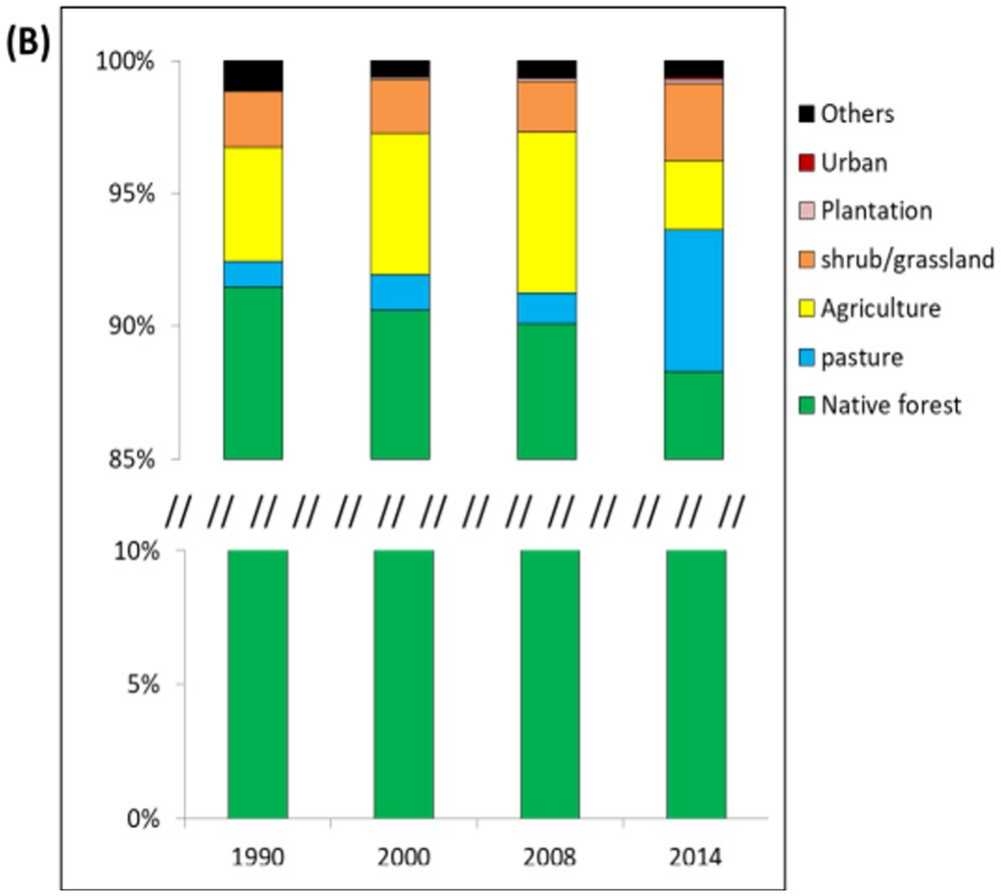
The major land use cover types inside 64 forest ecosystems in 1990, 2000, 2008 and 2014.

Despites its biological importance, recent data suggests a pessimistic future of biodiversity in Ecuador. According to the Food and Agriculture Organization of the United Nations [41], Ecuador has maintained the highest deforestation rates in South America at the country level during the last 20 years (annual rates of 1.5% and 1.8% for the periods 1990–2000 and 2000–2010, respectively). To date, agricultural expansion, wood extraction commercial logging, cacao and banana plantations, mining and road construction have been reported as the main drivers of ongoing land cover change in Ecuador (Fig 2) [31, 33].

**Fig 3:**
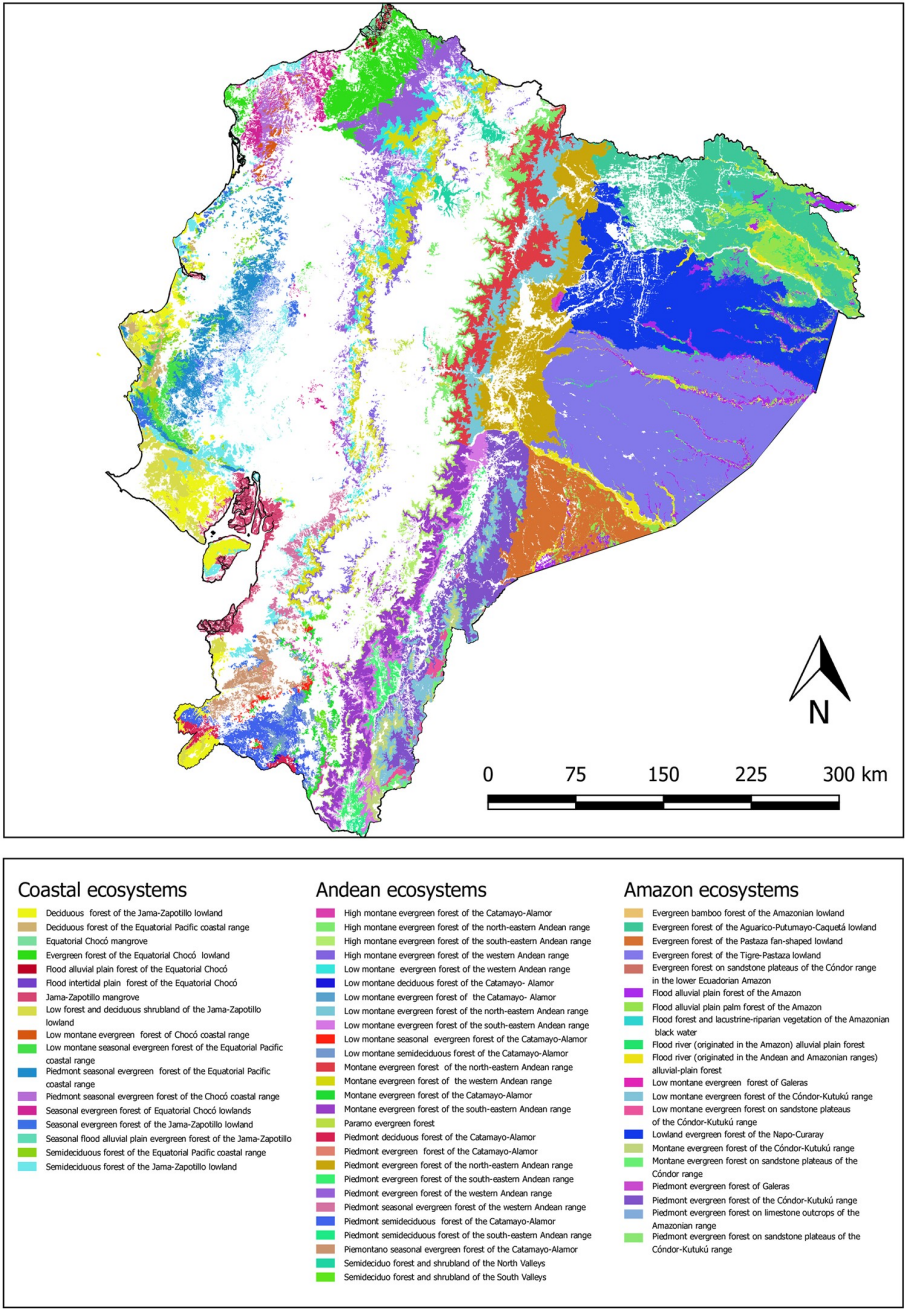
Distribution map of 64 forest ecosystems in Ecuador continent.

Ecosystem maps, which exhibit the spatial distribution of ecosystems, are the basis of assessing risk to ecosystems [42, 43]. In this study, we used the baseline information of ecosystem types on a national level, which were generated by the Ecuadorian Ministry of Environment (MAE). As the development of a global ecosystem conceptual framework typology to describe and classify ecosystems is currently underway, the Ecuadorian ecosystem map was produced by fusing data from the IUCN habitats classification scheme and available spatially explicit data [14, 44, 45]. Definition, classification and delimitation of 91 terrestrial ecosystems were established on the basis of the following factors: 1) physiognomy; 2) bioclimate; 3) biogeography; 4) geoform; 5) general flooding; 6) phenology; 7) bioclimatic soil; and 8) substratum [44, 45]. Among 91 terrestrial ecosystems within Ecuador, 89 correspond to natural ecosystems, with 64 forests, 13 grasslands and 12 shrublands. In the present study, we selected and analyzed the potential distribution of 64 forest ecosystems, including two mangrove ecosystems (Fig 3). The potential distributions of these forest ecosystems might include other types of land use and cover as a result of human-induced changes [44]. To apply criteria B, evidence of ongoing decline of an ecosystem type was assessed using the land use maps of 1990 and 2014 by [46] (Table 3).

### Framework of the assessment based on IUCN criteria

**Fig 4:**
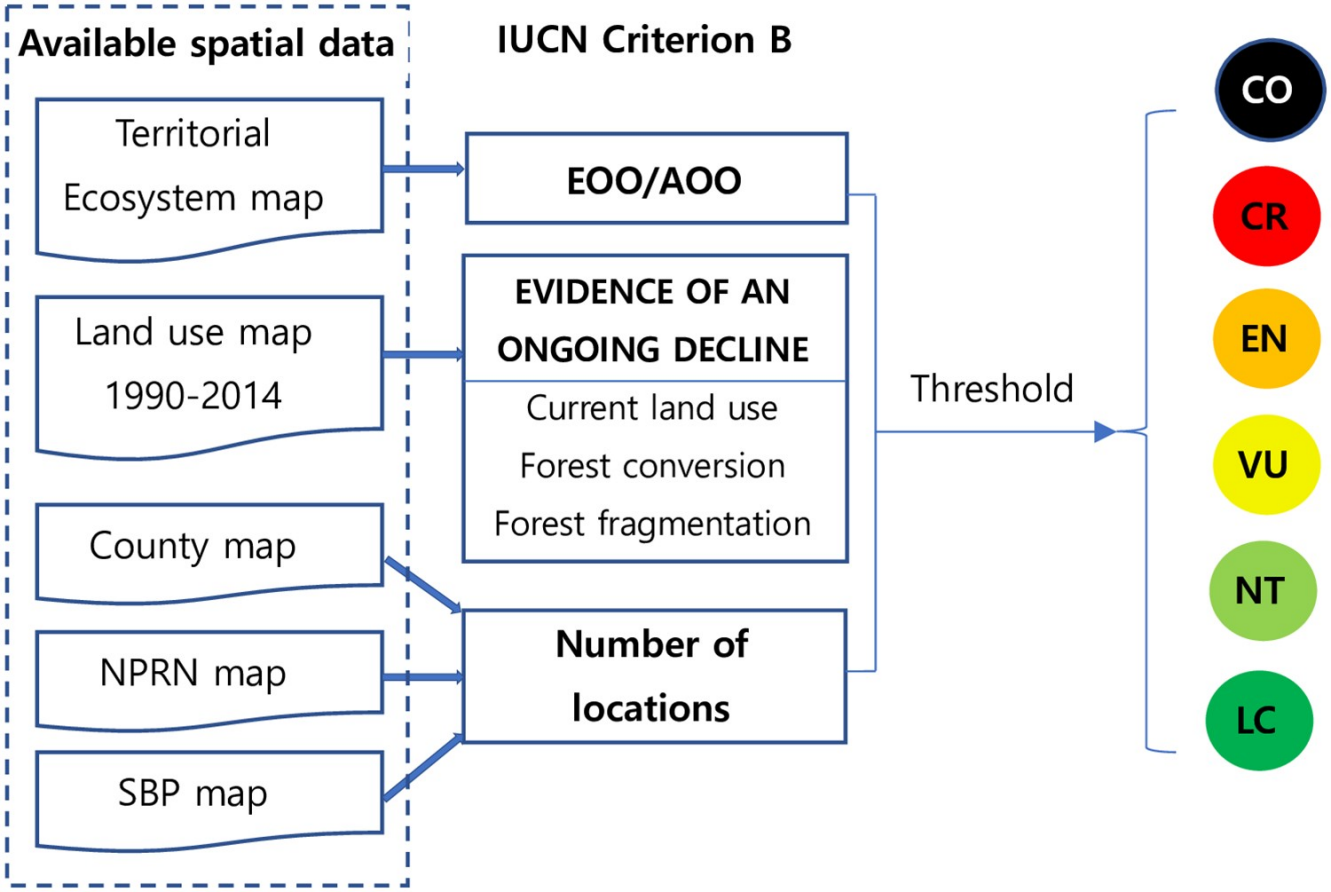
Available spatial data and workflow of assessing the risk to ecosystems in this study.

Due to the lack of available geospatial data across time for the application of criteria A, C and D, we only applied criteria B in this study. We show the workflow of assessing the risk to ecosystems in this study (Fig 4).

#### Assessment of criteria B

The current distribution of 64 forest ecosystems was quantified applying IUCN criteria B: the extent of geographic distribution of an ecosystem influences its risk of collapse when exposed to spatial threats [20]. To calculate the EOO and AOO of each ecosystem type, a 30 m grid scale ecosystem map [44] was used (Table 1).

**Table 1:** Summary of IUCN Red List criteria A and B for ecosystems V. 2.0 and sub-criteria applied for the present study.

Criteria B: restricted geographic distribution indicated by ANY of B1, B2 or B3		Critically Endangered (CR)	Endangered (EN)	Vulnerable (VU)
1	Extent of Occurrence (EOO) and observed or inferred continuing decline	≤2,000 km^2^	≤20,000 km^2^	≤50,000 km^2^
		At least one of the following:		
		(a) (i) a measure of spatial extent appropriate to the ecosystem OR ii) a measure of environmental quality appropriate to characteristic biota of the ecosystem OR iii) a measure of disruption to biotic interactions appropriate to the characteristic biota of the ecosystem.		
		(b) Observed or inferred threatening processes that are likely to cause continuing declines in geographic distribution, environmental quality or biotic interactions within the next 20 years.		
		(c) Ecosystem exits at		
		1 location	≤ 5 locations	≤ 10 locations
2	Area of Occurence (AOO) Number of 10x10 km grid cell and observed or inferred continuing decline	≤2	≤20	≤50
		At least one of the following: same as for B1.		
3	Number of locations	Very small (generally fewer than 5) AND prone to the effects of human activities or stochastic events within a very short time period in an uncertain future, and thus capable of collapse or becoming CR within a very short time period (B3 can only lead to a listing as VU).		

### Evidence of ongoing decline of an ecosystem

Spatial data describing current or potential threats to forest loss was obtained from a number of sources presented in Fig 4. Sub-criteria B1(a) and B2(a) address continuous declines in ecosystem distribution, abiotic environment or biotic processes. To capture threats applying sub-criteria B1(a)i or B1(a)ii, “Current land use”, “Forest conversion to cultivated area” and “Forest fragmentation” were considered to identify decline of spatial extent, based on 30 m grid land use maps in 1990 and 2014 [46].

#### Decline of spatial extent (B1ai OR B2ai)

As land use and cover have profoundly changed the natural habitats [47], we analyzed the current land use inside potential distribution of each forest ecosystem class, using five main land use types (Table 2) [46]: native forest, grassland/shrubland, agricultural land, urban area and other land cover (e.g., bare land and water bodies). Human-related land use types were considered as agricultural areas and urban areas. As a threat, severe human land use was defined as human land use > 40% of the total ecosystem area per ecosystem type in 2014.

**Table 2:** Land use and cover types that may be found within the potential distribution of forest ecosystem classes.

No.	Main land cover	No.	Subcategory
1	Native forest	1	Native forest
2	Grassland/shrubland	2	Grassland
		3	Shrubland
		4	Paramo
3	Agricultural area	5	Permanent
		6	Semi-permanent
		7	Annual
		8	Mixed
		9	Pasture
		10	Industrial plantation
4	Urban area	11	Inhabited area
		12	Infrastructure
5	Others	13	Natural water
		14	Artificial water
		15	Bare soil
		16	Glacier

Also, the conversion rate of native forest to agriculture, pasture and forest plantation within each ecosystem type was assessed using the land use maps of 1990 and 2014 of the Ministry of Agriculture, Livestock, Aquaculture and Fisheries (MAGAP) and the MAE [46], generated by LANDSAT 4 and 5 TM for 1990 and LANDSAT 8 OLI, LANDSAT ETM+, Rapid Eye satellite images for 2014. The thematic map of 2014 was classified by supervised classification using data from field surveys (at least 30 sites were monitored for each land use type). Meanwhile, the map for 1990 was generated by unsupervised classification [48, 49]. The agricultural land included permanent, semi-permanent, annual and mixed agriculture, industrial plantation and pasture (Table 2). “Severe forest conversion to cultivated land” was defined as at least 30% conversion of the forest ecosystem type to cultivated land between 1990 and 2014.

#### Decline of environmental quality to characteristic biota (B1aii OR B2aii)

As a measure of environmental quality to characteristic biota of an ecosystem, we analyzed forest fragmentation in each ecosystem. As fragmentation is summarizing a variety of spatial attributes of a forest, the analysis of forest fragmentation assessment was conducted using GUIDOS [50], which accounts for key aspects of fragmentation and multiple simultaneous effects, such as the area and shape of continuous forest, forest integrity (amount, shape, and area of perforations inside intact forests), and the spatial inter-patch distance distribution of forest patches separated by non-forest lands [51]. Vogt (50) reports the methodology to describe and quantify forest fragmentation and temporal change by measuring forest area density (FAD). The FAD values at 27 pixels-length scale are classified in two classes: separated (FAD < 40%) and continuous (40% ≤ FAD ≤ 100%). Ecosystems with low values of continuous FAD are subject to high level fragmentation. We assumed that ecosystem cover under 30% of continuous native forest was at high risk of forest fragmentation (i.e., “severe fragmentation”).

#### Number of locations (B1c OR B2c OR B3)

**Fig 5:**
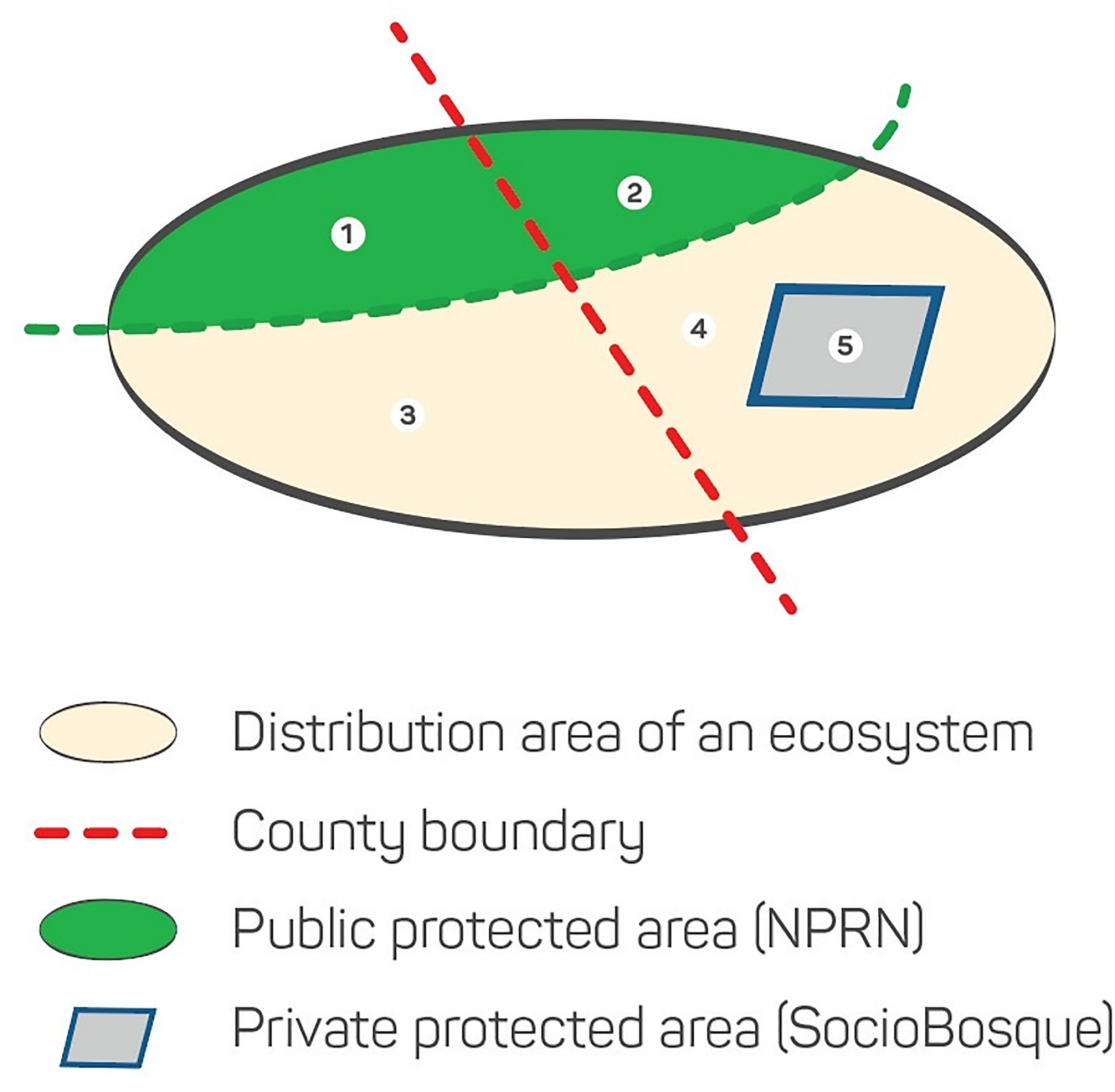
Method to quantify number of locations (for example, n = 5) within an ecosystem, using regulatory control on land use in the present study.

A location is defined as a geographically or ecologically distinct area in which a single threatening event can rapidly affect all occurrences of an ecosystem type [40]. As the most severe threat to the ecosystem in tropical landscapes is land transformation associated with agricultural expansion, the number of locations, therefore, determined using three jurisdictional zones with different regulatory controls on land use: i) county boundary, ii) public protected area, and iii) private protected area [20]. Data were derived from the National Parks and Reserves Network (NPRN) and Socio Bosque Program (SBP), which are managed by the Ministry of the Environment of Ecuador (MAE), as well as the county map from the National Mapping Agency (IGM) (Table 3). It was superimposed over the distribution map of forest ecosystems to generate the ecosystem extent incorporated with different land use control (Fig 5).

**Table 3:** Summary of data sources.

Name	Resolution	Source
**Territorial Ecosystem map**	30m	MAE^a^, 2013 [44]
**Land use maps**	30m	MAGAP^b^-MAE 1990, 2014 [46]
**County map**	30m	IGM^c^, 2018 [52]
**NPRN**^d^ **map**	30m	MAE, 2018 [48]
**SBP**^e^ **map**	30m	MAE, 2012 [48]

^a^MAE = Ministry of Environment;

MAGAP = Ministry of Agriculture, Livestock, Aquaculture and Fisheries;

^c^IGM = National Mapping Agency;

^d^NPRN = National Parks and Reserves Network;

^e^SBP = Socio Bosque Program.

## Results

### Identification of spatially restricted forest ecosystems

We identified 60 ecosystems with restricted EOO (11 ecosystems of EOO ≤ 2,000 km^2^, 30 of > 2,000 and ≤ 20,000 km^2^ and 19 of > 20,000 and ≤ 50,000 km^2^) and 28 ecosystems with restricted AOO (4 ecosystems of AOO ≤ 2, 15 of > 2 and ≤ 20, and 9 of > 20 and ≤ 50). A total of 28 ecosystems were classified as restricted geographic distributions indicated by either EOO or AOO (Table 4).

**Table 4:** List of 64 terrestrial forest ecosystems in Ecuador (S1 Table), assessed by IUCN RLE criteria B.

Ecosystem code	Criteria B		Sub-criteria assessed (● detected evidence of decline, ○ assessed but no evidence found)				Criteria determining overall status	IUCN status
			(a)i		(a)ii	(c)		
	EOO(km2)	AOO (# 10 km x 10 km)	Current human land use^1)^	Conversion to agriculture^2)^	Forest fragmentation^3)^	No. location^4)^		
**E01**	3.32	1	○	○	○	**2**	B1, B2(c)	EN
**E02**	6,338.54	26	○	○	○	67	B1, B2	NT
**E03**	22,444.80	100	○	○	○	429	B2, B3	LC
**E04**	679.66	14	○	○	○	36	B1, B2	NT
**E05**	20,512.71	75	○	○	○	107	B2, B3	LC
**E06**	4,476.46	53	○	○	○	69	B1, B2	LC
**E07**	2,877.61	34	○	○	○	49	B1, B2	NT
**E08**	54,091.38	216	○	○	○	341	B1, B2	LC
**E09**	27,147.75	107	○	○	○	150	B1, B2	LC
**E10**	32,207.88	91	○	○	●	158	B1(a)ii	VU
**E11**	23,261.57	148	○	○	○	225	B1, B2	LC
**E12**	23,185.29	84	○	○	○	168	B1, B2	LC
**E13**	47,546.70	122	●	●	○	157	B1(a)i	VU
**E14**	45,460.15	153	○	○	○	306	B1, B2	LC
**E15**	10,183.20	40	○	○	○	93	B1, B2	NT
**E16**	22,742.13	79	○	○	○	45	B1, B2	LC
**E17**	193.53	4	●	●	●	**8**	B1(a)i,ii	CR
**E18**	27,846.44	173	○	○	○	724	B1, B2	LC
**E19**	31,046.19	171	○	○	○	519	B1, B2	LC
**E20**	36,784.07	193	○	○	○	695	B1, B2	LC
**E21**	33,510.24	146	○	○	○	457	B1, B2	LC
**E22**	8,130.38	43	○	○	○	49	B1, B2	NT
**E23**	4,333.59	48	●	●	○	192	B1(a)i	EN
**E24**	3,763.32	20	○	○	○	60	B1, B2	NT
**E25**	8,325.38	57	○	○	○	153	B1, B2, B3	LC
**E26**	4,087.80	20	○	○	○	121	B1, B2	NT
**E27**	547.67	10	●	○	○	54	B1(a)i	CR
**E28**	125.85	3	○	○	○	11	B1, B2, B3	NT
**E29**	6,123.16	65	○	○	○	155	B1, B2	LC
**E30**	3,657.26	44	○	○	○	72	B1, B2	LC
**E31**	2,660.12	20	○	○	○	62	B1, B2	NT
**E32**	184.98	2	●	●	●	**6**	B1, B2(a)i,ii	CR
**E33**	1,012.12	11	○	○	●	81	B1(a)ii	CR
**E34**	2,069.48	9	○	●	●	17	B1, B2(a)i,ii	EN
**E35**	8,870.84	13	○	○	●	164	B1, B2(a)ii	EN
**E36**	20,023.85	139	○	○	○	382	B1, B2	LC
**E37**	21,641.19	152	○	○	○	391	B1, B2	LC
**E38**	15,052.86	130	○	○	○	527	B1, B2	LC
**E39**	18,298.44	158	○	○	○	657	B1, B2	LC
**E40**	15,732.84	127	○	○	○	286	B1, B2	LC
**E41**	18,133.01	165	○	○	○	305	B1, B2	LC
**E42**	14,426.60	125	○	○	○	136	B1, B2	LC
**E43**	12,877.16	89	○	○	○	221	B1, B2	LC
**E44**	193.80	4	●	●	●	**5**	B1(a)i,ii	CR
**E45**	39.34	2	○	○	○	**9**	B1, B2(c)	VU
**E46**	135.55	3	○	○	○	16	B1,B2	NT
**E47**	17,786.25	152	○	○	○	280	B1, B2	LC
**E48**	13,552.64	121	○	○	○	195	B1, B2	LC
**E49**	10,581.21	73	○	○	○	144	B1, B2	LC
**E50**	8,094.90	26	○	○	○	22	B1, B2	LC
**E51**	4,297.49	20	○	○	○	47	B1, B2	NT
**E52**	6,523.91	23	○	○	○	37	B1, B2	NT
**E53**	5,837.63	35	○	○	○	99	B1, B2	LC
**E54**	18.79	1	○	○	○	**2**	B1, B2(c)	EN
**E55**	23,716.19	227	○	○	○	660	B1, B2	LC
**E56**	64,968.60	424	○	○	○	720	B1, B2	LC
**E57**	70,216.35	194	○	○	○	186	B1, B2	LC
**E58**	25,123.32	237	○	○	○	411	B1, B2	LC
**E59**	55,127.77	316	○	○	○	241	B1, B2	LC
**E60**	5,256.45	14	○	○	○	25	B1, B2	NT
**E61**	43,108.96	153	○	○	○	59	B1, B2	LC
**E62**	4,372.73	12	○	○	○	**6**	B1, B2(c)	VU
**E63**	26,774.64	289	○	○	○	1,320	B1, B2	LC
**E64**	8,822.89	98	○	○	○	40	B1, B2	LC

EOO = extent of occurrence, AOO = area of occurrence, CR = critically endangered, EN = endangered, VU = vulnerable, LC = least concern, NT = near threatened.

^1)^ Agriculture + urban area > 40% of total ecosystem area in 2014.

^2)^ Conversion of forest to cultivated land > 30% between 1990 and 2014.

^3)^ Continuous native forest < 30% of ecosystem cover in 2014.

^4)^ Number of geographically distinct area using three regulatory controls: county boundary, public protected area and private protected area.

### Potential threats of forest ecosystem collapse

Total 14 forest ecosystems were associated to at least one of potential threats of ecosystem collapse. In most of the forest ecosystems, evidence on ongoing decline or very few locations were not observed (Table 4).

#### Current land use

**Fig 6:**
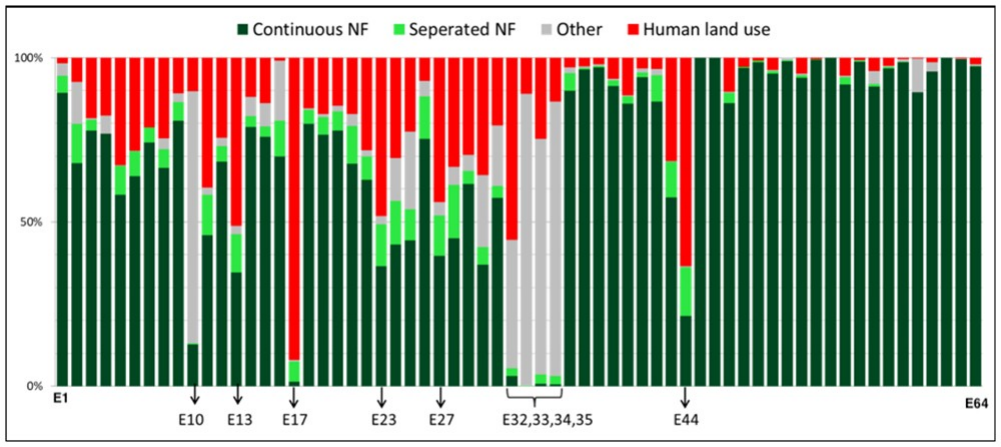
The major land cover types of single ecosystems (n = 64) in 2014. Continuous and separated native forests were distinguished based on the Forest Area Density (FAD) values calculated from GUIDOS. Human land use and cover includes agricultural land, pasture, forest plantation and urban area. Ecosystems containing either continuous native forests <30% or human land use >40% (n = 5) are E 10, 13, 17, 23, 32, 33, 34, 35 and 44.

In 2014, many forest ecosystems located on the coast, western Andes and valley were affected by direct human activities (Fig 6). For example, native forest remained only 7.6% in “seasonal flood alluvial plain evergreen forest of the Jama-Zapotillo (E17)” in a landscape dominated by human land use. Across the entire country, the primary form of land use change in the forest system was the conversion to pastures (45.67% of converted area), followed by natural shrub/grassland (24.77%), agricultural land (22.31%), others (5.48%), industrial plantation (1.18%) and urban (0.60%) (Fig 6). Based on the definition of “severe human land use”, it was found that six forest ecosystems have shown strong effects on human activities in 2014 (Fig 6).

#### Conversion to cultivated land

Forest conversion rate to cultivated land between 1990 and 2014 ranged from 0.5% to 98.9% in ecosystems located on the coast, between 0.7% and 60% in the Andes, and between 0% to 4% in the Amazon. Forests were not converted to any type of cultivated land in three forest ecosystems in the Amazon: E52, E55 and E64. Conversely, six forest ecosystems were classified as severe conversion to cultivated land: E17 (98.8%), E44 (60%), E23 (36.8%), E34 (34.4%), E32 (33.4%) and E13 (31.6%).

#### Forest fragmentation

As evidence of the decline of environmental quality to characteristic biota, the analysis of forest fragmentation showed the distribution of continuous forests across the potential limits of 64 forest ecosystem classes (Fig 6). We mainly distinguished seven ecosystems in severe forest fragmentations (continuous native forests of ≤ 30% within an ecosystem): E10, E17, E32, E33, E34, E35 and E44.

#### Number of locations

According to the estimated number of locations that are occupied relative to the extent of serious plausible threat of land use change, we identified seven ecosystems in 10 locations: E1 (2 locations), E17 (8), E32 (6), E44 (5), E45 (9), E54 (2) and E62 (6).

**Fig 7:**
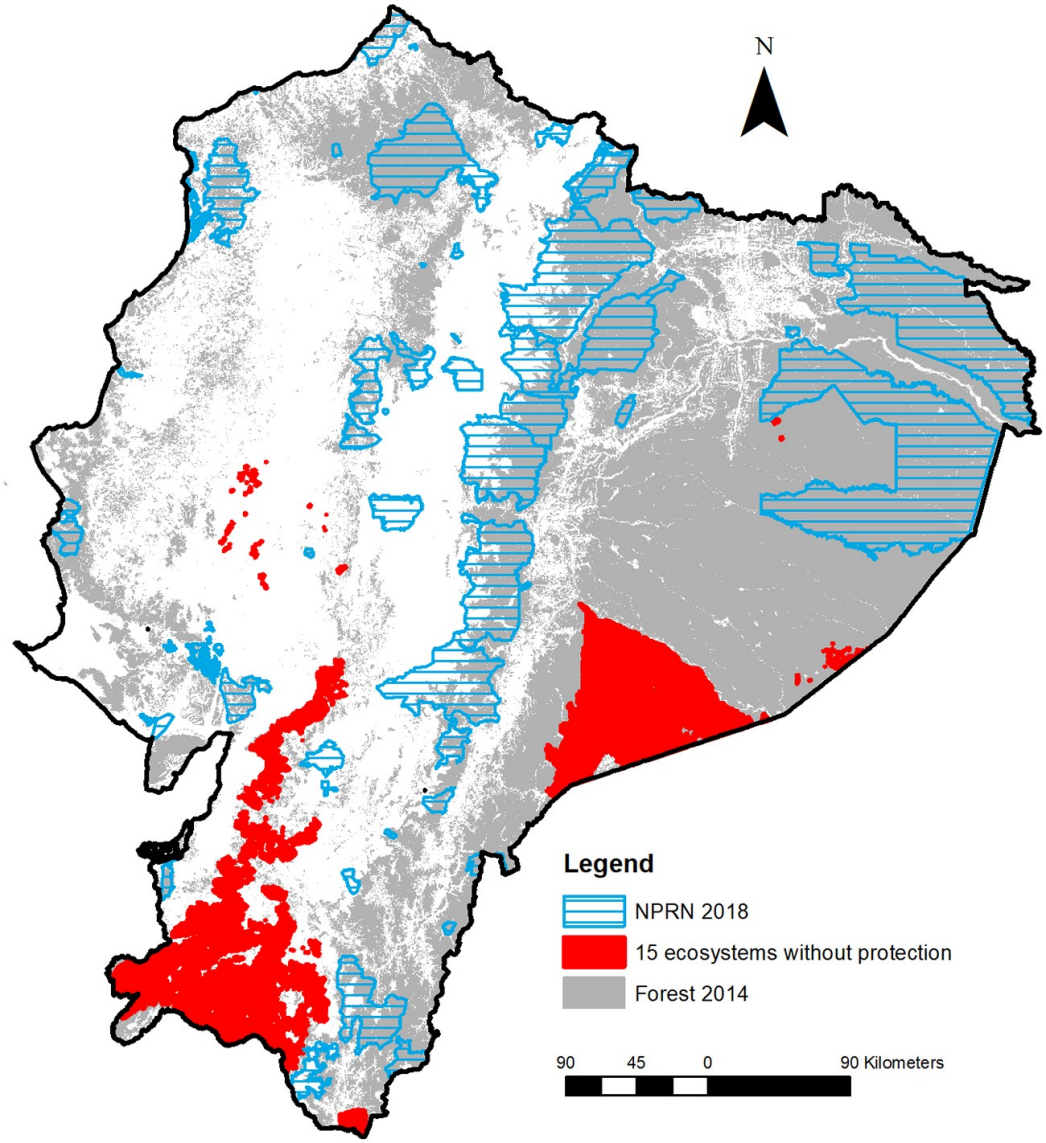
Distribution map of 15 forest ecosystems without National Parks and Reserves Network (NPRN).

In 2014, the percentage of protected areas in each ecosystem varied (SD = 28.3, range 0–100). Only in the case of E45, the entire land extent was under protection. A total of 34 forest ecosystems (5 ecosystems on the coast, 16 in the Andes and 13 in the Amazon) were identified as having less than 17% of their protected area. Among them, 15 ecosystems without national protection were E17, E22, E23, E24, E27, E28, E29, E30, E31, E32, E34, E44, E54, E62 and E64 (Fig 7). Also, calculating the difference between the proportion of native forest and protected land in a given ecosystem, deforestation within the protected areas was observed in 4 forest systems: E11, E17, E24 and E32. An example is E17, which showed only 7.4% of native forest in a landscape under 59.75% of land protection in 2014.

**Fig 8:**
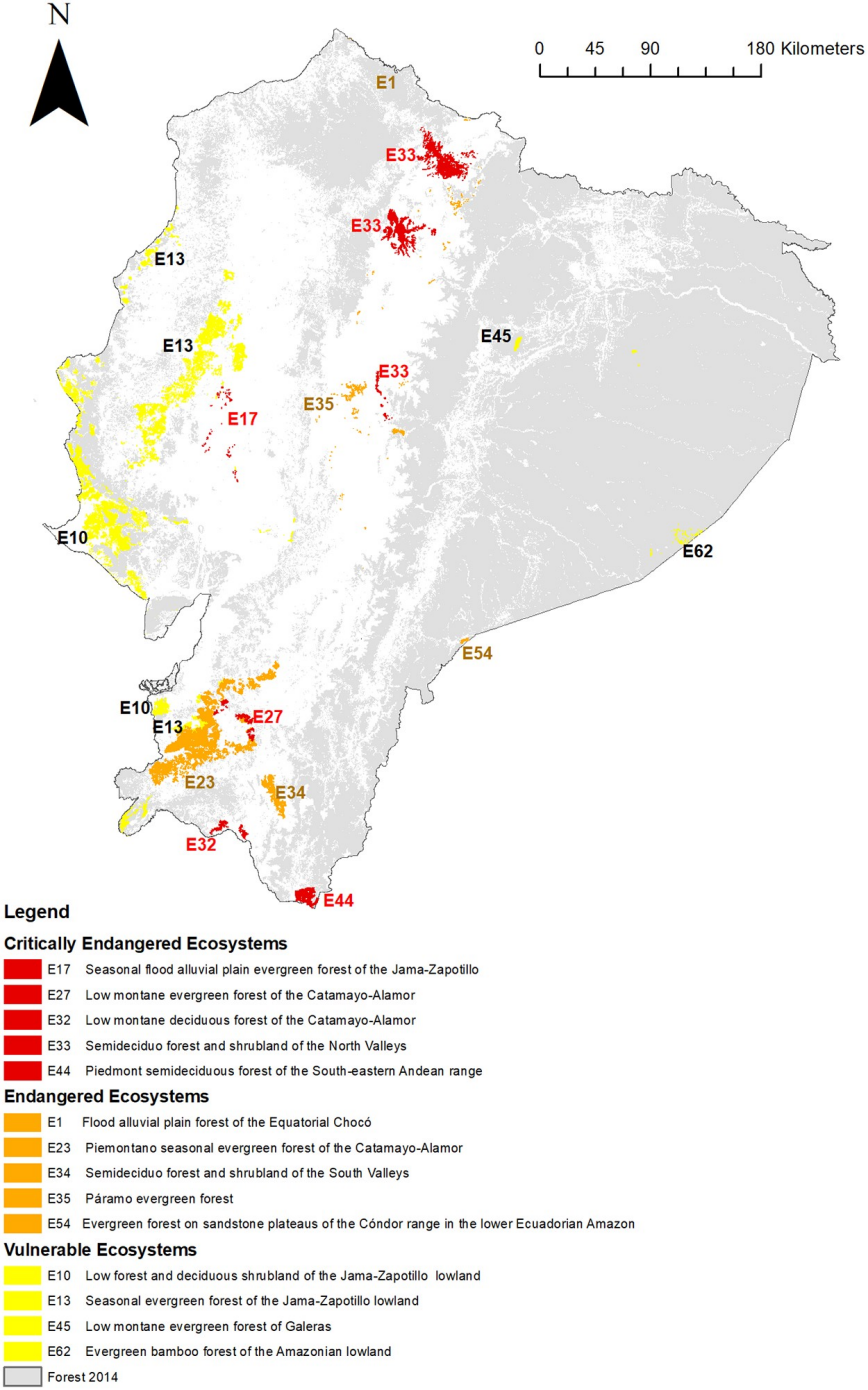
Map of 13 threatened forest ecosystems of the Ecuadorian mainland; assessed by IUCN.

Our results revealed that 14 ecosystems were threatened (Fig 8, Table 4): five were categorized by IUCN RLE as critically endangered (E17, E27, E32, E33, and E44), five as endangered (E1, E23, E34, E35, and E54) and four as vulnerable (E10, E13, E45, and E62), which represented 22% of the total forest ecosystems and 2.95% (≈ 4,005.84 km^2^) of the total area of forest ecosystems within Ecuador.

## Discussion

Many forest ecosystems in Ecuador suffer from human activities and pressure, particularly in the sub-montane area. However, it is difficult to precisely assess the local extent of such pressures in terms of their effects on structure and composition or disappearance of the ecosystems. Our most important, but perhaps least surprising, result is that many tropical Andean forest systems are facing extinction risk on both national and local levels in Ecuador. In the present study, we estimated that several tropical Andean forest systems are rapidly changing and probably disappearing faster than other forest ecosystems. The results suggest that the success of ecosystem conservation will increase with the merits of a conservation prioritization system based on the ecological and biogeographical knowledge of the ecosystem [53, 54].

As knowledge of biogeographic zoning at the national level is rarely available, previous studies about RLE focused on single territorial ecosystems, small areas or regions reported and assessed a probability of loss or degradation. In northern Venezuela, Rodríguez, Nassar (24) assessed the extinction risk categories of tropical dry forests using historical and current dry forest cover. Likewise, the conservation status of temperate grasslands in southern Africa was estimated by the combination of two landscape-scale factors: level of protection and degree of land transformation [55]. However, these results provided limited information to identify the critical areas for shaping national conservation policy. Likewise, methods for assessing the threat of extinction of individual ecosystems were not systematized in many of the previous studies. For example, although Sierra, Campos (9) identified the prioritization among 46 natural ecosystems for the conservation of Ecuador´s biodiversity using a multi-criteria model, their criteria (representativeness in the current reserve network, human pressure, habitat loss and species-level value based on bird species data) associated with the developed model were not directly linked with key symptoms of ecosystem degradation. Therefore, unlike the results of Sierra, Campos (9) who found 26 critical ecosystems, we identified 14 threatened ecosystems: four on the coast, seven in the Andes and three in the Amazon.

A central benefit of assessing the conservation status of nationwide ecosystems from a systematic method is that policymakers may become explicitly aware of the spatial scale at which their policies are implemented or affected between conservation and development of a given ecosystem. Forest ecosystem change has been particularly severe in tropical regions of developing countries under the pressure of strong socioeconomic drivers [56–58]. To mitigate the dramatic deforestation rate of the country, the Ecuadorian government has promoted incentive-based policies for the conservation of native forests, such as the Socio Bosque program [59] as well as the establishment of several protected areas [29]. From a conservation point of view, there are two concerns with regard to existing forest protection policies. The first one is that the NPRN in Ecuador is not optimized for the protection of natural forest ecosystems, despite the protected areas seem to be effective for avoiding or reducing deforestation in Ecuadorian tropical Andean forests [29]. Although threatened or near threatened forest ecosystems are concentrated in southern Ecuador, we demonstrated that the current NPRN coverage does not provide an appropriate protection for these critical ecosystems. Secondly, the strategy of protected areas is not considered to effectively expand conservation areas by connecting isolated areas of important ecosystems or habitats outside of protected areas [59]. Therefore, stakeholders and funding agencies are questioning the effectiveness and efficiency of nationwide ecosystem conservation, although private and community land owners can benefit from a financial incentive in exchange for conservation of forests through the Socio Bosque Program [47, 60].

In Ecuador, it seems that conservation policies to avoid forest loss, such as Socio Bosque, REDD+, NPRN, water founds, among others, have had a presumably positive effect on spatial extent and environmental quality of forest ecosystems [29, 59]. Our study demonstrated that many forest ecosystems in Ecuador mainland do not represent an evidence on ongoing decline despite their restricted distribution, being them possible to classify less threat level (Table 4). The main challenge for future forest ecosystem conservation is a lack of explicit policies for management and use. We observed a lack of protection in threatened or near-threatened forest ecosystems, which may result in conservation gaps for species and ecosystems in the country [61]. Although establishing new areas under protection might be a long and difficult process due to conflicts with relevant stakeholders [62], the role of specific forest ecosystems based on ecosystem services might support political, social, and economic justifications based on a contribution to human well-being [6]. Recognizing the demand and provision of ecosystem services that are supplied by a locally-threatened ecosystem may promote informed decisions regarding investments in ecosystem protection and restoration [24]. Aiming to strengthen conservation, valorization and sustainable use of natural resources, ecosystem services and biodiversity, ecosystem conservation strategies may be designed to further achieve environmental sustainability and territorial development.

Our analyses provide the first potential evidence of future loss of tropical Andean ecosystems in the tropical Andean biodiversity hotspot according to the IUCN RLE criteria. Specific recommendations and more detailed future field studies for the management of these threatened or near-threatened ecosystems should include: (1) restoring forest quality and mitigating the trend toward a loss and degradation of ecosystems; (2) creating buffers around remaining forests, in order to reduce edge effects and improve landscape connectivity; (3) future research on adaptive capacity of the threatened ecosystems with regard to anthropogenic (e.g., logging, agriculture, and fragmentation) and intrinsic (e.g., forest fire, flooring, and climate change) threats and their interactions; (4) research to determine the threshold of resilience and vulnerability of the remaining forest patches in each ecosystem; (5) promoting off-reserve conservation on privately or communally owned lands; and (6) identifying and designing adequate landscape configuration based on the remnant forests in order to enhance ecosystem persistence and resilience [63, 64]. One of the limitations of this study was the mapping of intrinsic threats restricted to the scale (30 m resolution).

## Conclusions

The present analysis of conservation status of forest ecosystems in Ecuador drew several conclusions: i) only a small extent of forest patches remained in several forest systems; ii) these forest systems are at risk of extinction due to pressure from human land use, and iii) the management by official institutions could be improved with respect to the protection of forest ecosystems. This study stands as the baseline for the identification and understanding of forest ecosystem change, threats and potential extinction risk at a landscape scale. It could complement current conservation efforts and contribute to guide land use planning at local and national levels in the mainland of Ecuador.

## Supporting information

S1 Table Spatial scale (nation-region-ecoregion-ecosystem) of the system under study and 64 forest ecosystems of continental Ecuador. (DOCX)
