# Supplementary material for: Warning about conservation status of forest ecosystems in tropical Andes: National assessment based on IUCN criteria
Source: PLoS One. 2020 Aug 25;15(8):e0237877. doi: 10.1371/journal.pone.0237877 (PMC7447026; doi:10.1371/journal.pone.0237877)
Supplement: S1 Table — (DOCX) [file pone.0237877.s001.docx]

**S1 Table.**  **Spatial scale (nation-region-ecoregion-ecosystem) of the system under study and 64 forest ecosystems of continental Ecuador.**

| **Region** | **Ecoregion** | **Code** | **Forest Ecosystems (forest vegetation type)** | **Altitudinal range (m)** |
| --- | --- | --- | --- | --- |
| **COAST** | Equatorial-Chocó | E1 | Flood alluvial plain forest of the Equatorial Chocó | 50-200 |
|  |  | E2 | Equatorial Chocó mangrove | 0-20 |
|  |  | E3 | Evergreen forest of the Equatorial Chocó lowland | 0-300 |
|  |  | E4 | Flood intertidal plain forest of the Equatorial Chocó | 0-50 |
|  |  | E5 | Seasonal evergreen forest of Equatorial Chocó lowlands | 0-300 |
|  |  | E6 | Piedmont seasonal evergreen forest of the Chocó coastal range | 200-400 |
|  |  | E7 | Low montane evergreen forest of Chocó coastal range | >400 |
|  | Equatorial-Pacific | E8 | Semideciduous forest of the Jama-Zapotillo lowland | 0-300 |
|  |  | E9 | Semideciduous forest of the Equatorial Pacific coastal range | >200 |
|  |  | E10 | Low forest and deciduous shrubland of the Jama-Zapotillo lowland | 0-400 |
|  |  | E11 | Piedmont seasonal evergreen forest of the Equatorial Pacific coastal range | 200-400 |
|  |  | E12 | Low montane seasonal evergreen forest of the Equatorial Pacific coastal range | 400-860 |
|  |  | E13 | Seasonal evergreen forest of the Jama-Zapotillo lowland | 0-400 |
|  |  | E14 | Deciduous forest of the Jama-Zapotillo lowland | 0-400 |
|  |  | E15 | Deciduous forest of the Equatorial Pacific coastal range | >200 |
|  |  | E16 | Jama-Zapotillo mangrove | 0-10 |
|  |  | E17 | Seasonal flood alluvial plain evergreen forest of the Jama-Zapotillo | 0-300 |
|  |  |  |  |  |
| **ANDES** | Western-range | E18 | Piedmont evergreen forest of the western Andean range | 300-1400 |
|  |  | E19 | Low montane evergreen forest of the western Andean range | 1400-2000 |
|  |  | E20 | Montane evergreen forest of the western Andean range | 2000-3100 |
|  |  | E21 | High montane evergreen forest of the western Andean range | 3100-3600 |
|  |  | E22 | Piedmont seasonal evergreen forest of the western Andean range | 300-1400 |
|  |  | E23 | Piemontano seasonal evergreen forest of the Catamayo-Alamor | 400-1600 |
|  |  | E24 | Low montane seasonal evergreen forest of the Catamayo-Alamor | 1600-2000 |
|  |  | E25 | Montane evergreen forest of the Catamayo-Alamor | 2200-2900 |
|  |  | E26 | High montane evergreen forest of the Catamayo-Alamor | 2900-3400 |
|  |  | E27 | Low montane evergreen forest of the Catamayo- Alamor | 1600-2200 |
|  |  | E28 | Piedmont evergreen forest of the Catamayo-Alamor | 400-1600 |
|  |  | E29 | Piedmont semideciduous forest of the Catamayo-Alamor | 400-1600 |
|  |  | E30 | Low montane semideciduous forest of the Catamayo-Alamor | 1600-2200 |
|  |  | E31 | Piedmont deciduous forest of the Catamayo-Alamor | 400-1600 |
|  |  | E32 | Low montane deciduous forest of the Catamayo- Alamor | 1600-2200 |
|  | Valley, Alpine | E33 | Semideciduo forest and shrubland of the North Valleys | 1200-2600 |
|  |  | E34 | Semideciduo forest and shrubland of the South Valleys | 1200-2000 |
|  |  | E35 | Páramo evergreen forest | 3200-4100 |
|  | Eastern-range | E36 | High montane evergreen forest of the north-eastern Andean range | 3000-3700 |
|  |  | E37 | Montane evergreen forest of the north-eastern Andean range | 2000-3000 |
|  |  | E38 | Low montane evergreen forest of the north-eastern Andean range | 1200-2000 |
|  |  | E39 | Piedmont evergreen forest of the north-eastern Andean range | 400-1200 |
|  |  | E40 | Low montane evergreen forest of the south-eastern Andean range | 1660-2200 |
|  |  | E41 | Montane evergreen forest of the south-eastern Andean range | 2200-3000 |
|  |  | E42 | High montane evergreen forest of the south-eastern Andean range | 3000-3400 |
|  |  | E43 | Piedmont evergreen forest of the south-eastern Andean range | 400-1650 |
|  |  | E44 | Piedmont semideciduous forest of the south-eastern Andean range | 500-1300 |
|  |  |  |  |  |
| **AMAZON** | Range | E45 | Low montane evergreen forest of Galeras | 1300-1700 |
|  |  | E46 | Piedmont evergreen forest of Galeras | 600-1300 |
|  |  | E47 | Piedmont evergreen forest of the Cóndor-Kutukú range | 350-1400 |
|  |  | E48 | Low montane evergreen forest of the Cóndor-Kutukú range | 1400-1900 |
|  |  | E49 | Montane evergreen forest of the Cóndor-Kutukú range | 1900-2400 |
|  |  | E50 | Piedmont evergreen forest on sandstone plateaus of the Cóndor-Kutukú range | 350-1400 |
|  |  | E51 | Montane evergreen forest on sandstone plateaus of the Cóndor range | 1900-2700 |
|  |  | E52 | Piedmont evergreen forest on limestone outcrops of the Amazonian range | 600-1400 |
|  |  | E53 | Low montane evergreen forest on sandstone plateaus of the Cóndor-Kutukú range | 1400-1900 |
|  |  | E54 | Evergreen forest on sandstone plateaus of the Cóndor range in the lower Ecuadorian Amazon | 243-550 |
|  | Plain | E55 | Evergreen forest of the Aguarico-Putumayo-Caquetá lowland | 168-350 |
|  |  | E56 | Flood alluvial plain palm forest of the Amazon | 171-350 |
|  |  | E57 | Flood river (originated in the Andean and Amazonian ranges) alluvial-plain forest | 164-350 |
|  |  | E58 | Lowland evergreen forest of the Napo-Curaray | 170-350 |
|  |  | E59 | Flood alluvial plain forest of the Amazon | 158-350 |
|  |  | E60 | Flood forest and lacustrine-riparian vegetation of the Amazonian black water | 170-350 |
|  |  | E61 | Flood river (originated in the Amazon) alluvial plain forest | 158-350 |
|  |  | E62 | Evergreen bamboo forest of the Amazonian lowland | 196-500 |
|  |  | E63 | Evergreen forest of the *Tigre-Pastaza* lowland | 166-350 |
|  |  | E64 | Evergreen forest of the *Pastaza* fan-shaped lowland | 197-350 |
